# ENHANCING AND SUSTAINING THE MEMORY CARE NAVIGATOR MODEL IN HAWAII

**DOI:** 10.1093/geroni/igae098.1690

**Published:** 2024-12-31

**Authors:** Christy Nishita

**Affiliations:** University of Hawaii, Honolulu, Hawaii, United States

## Abstract

Hawaii’s memory care navigator model aims to educate, support, and connect local families to the resources that they need. The Kupuna Collective, a cross-sector coalition led by the University of Hawaii Center on Aging and Hawaii Public Health Institute, has taken steps to enhance the model. First, community health workers, given their close ties and relationship with local communities were targeted and trained to provide person-centered, dementia support in a culturally tailored manner. To ensure quality and explore collective impact, community health workers, who were embedded within local organizations, use a standardized, electronic platform to collect assessment and care planning data. In addition, the model has been sustained by integrating the model within Hawaii’s workforce development efforts. Trainings are widely disseminated as part of Hawaii’s Building Our Largest Dementia Infrastructure initiative, funded by the Centers for Disease Control. Trainings are stored in an online library for ongoing continuing education and training and available for use by community college CHW training programs. Taken together, the memory care navigator model has supported the development of a more coordinated system of dementia care in Hawaii.

